# Solution of magnetohydrodynamic flow and heat transfer of radiative viscoelastic fluid with temperature dependent viscosity in wire coating analysis

**DOI:** 10.1371/journal.pone.0194196

**Published:** 2018-03-29

**Authors:** Zeeshan Khan, Muhammad Altaf Khan, Nasir Siddiqui, Murad Ullah, Qayyum Shah

**Affiliations:** 1 Department of computer Science, Sarhad University of Science and Information Technology, Peshawar, Pakistan; 2 Department of Mathematics, City University of Science and IT, Peshawar, Pakistan; 3 Department of Basic Sciences, University of Engineering and Technology, Taxila, Pakistan; 4 Department of Mathematics, Islamia College University of Peshawar, Khyber Pakhtunkhwa, Pakistan; 5 Department of Basic Science and Islamiat, University of Engineering and Technology Peshawar, Khyber Pakhtunkhwa, Pakistan; Universitty of Newcastle, AUSTRALIA

## Abstract

Wire coating process is a continuous extrusion process for primary insulation of conducting wires with molten polymers for mechanical strength and protection in aggressive environments. In the present study, radiative melt polymer satisfying third grade fluid model is used for wire coating process. The effect of magnetic parameter, thermal radiation parameter and temperature dependent viscosity on wire coating analysis has been investigated. Reynolds model and Vogel’s models have been incorporated for variable viscosity. The governing equations characterizing the flow and heat transfer phenomena are solved analytically by utilizing homotopy analysis method (HAM). The computed results are also verified by ND-Solve method (Numerical technique) and Adomian Decomposition Method (ADM). The effect of pertinent parameters is shown graphically. In addition, the instability of the flow in the flows of the wall of the extrusion die is well marked in the case of the Vogel model as pointed by Nhan-Phan-Thien.

## Introduction

Investigation on the boundary-layer behavior of a viscoelastic fluid over a continuously stretching surface has many important applications in the extrusion of polymers, the treatment of plastic films and applications. Increasingly important applications of these industrial processes have led to renewed interest in the study of viscous fluid flows and heat transfer in the coating process. Metal coating is an industrial process for insulation isolation, environmental protection, mechanical deterioration and protection against signal attenuation. The simple and suitable process for wire coating is the coaxial extrusion process [1–4]. In wire coating, wire drawing rate, temperature and quality of materials are important parameters. Different types of fluids are used for coating which depends on die geometry, fluid viscosity, temperature, and molten polymer. Considerable attention has been paid to the Newtonian fluid to investigate the heat transfer analysis effect. However, less attention has been given to the study of non-Newtonian fluids [5–10]. However, some studies are listed to illustrate broader research [11–18].

**Fig 1:**
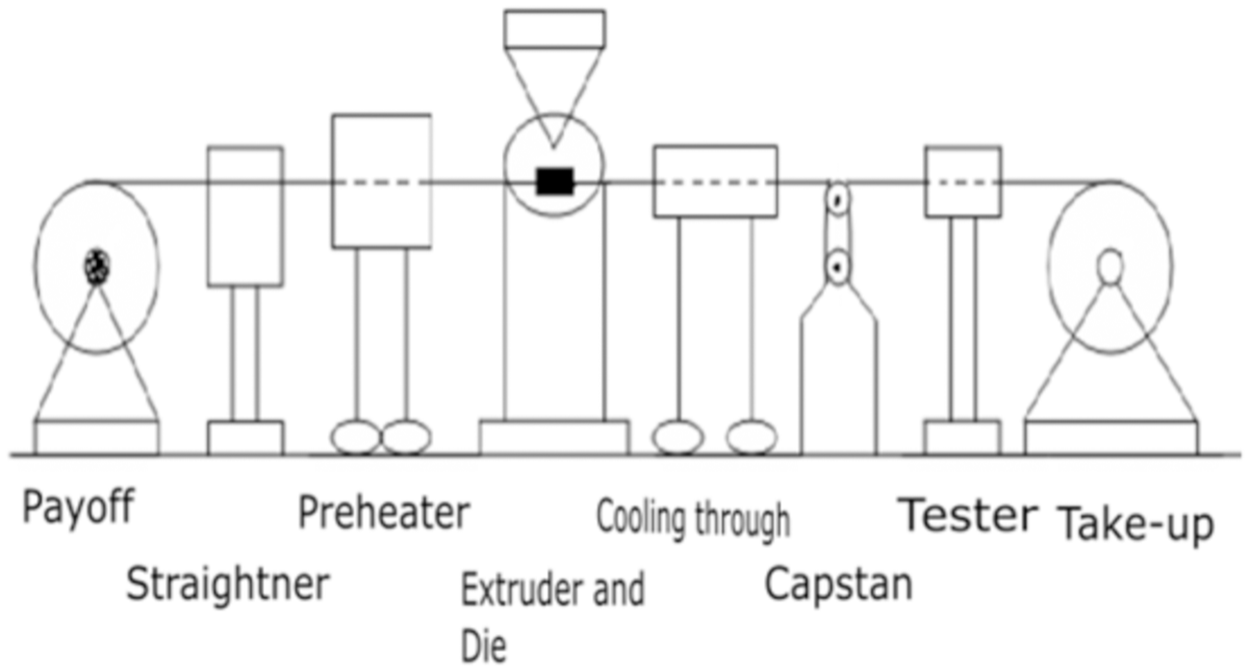
Typical wire coating process.

Fig 1 shows the experimental setup of the wire coating process [19]. In this process, the uncoated film comes off through a payoff reel through a straightener, a preheater, then it meets the polymer comes out of the extruder and gets coated.

This coating then passes through a cooler, a capstan and a test device and ends with the rotating device. The extrusion process is simple to apply, time saving and economical for industrial applications. Fata et al. [20], Siddiqui et al. [21], Tadmor and Gogod [22] used the extrusion process using third grade fluid for coating wires. Third grade fluid consider here used a coating material have industrial importance.

Hayat et al. [23] studied an axisymmetric MHD flow of a third grade fluid by a drawing cylinder. MHD unsteady flow of a third grade fluid has been investigated by Shuaib et al. [24] through a vertical belt. The hydromagnetic flow of a third grade fluid has been studied by Chinyoka and Makinde [25] with variable viscosity.

In manufacturing process final product greatly depend on the cooling rate. The central cooling system is beneficial to facilitate the process for the additional product. An electro conductive polymeric liquid seems to be a good candidate for industrial applications like in polymerization technology and extrusion processes, as the flow can be regulated by the magnetic field as well as porous matrix. The porous medium and the applied magnetic field can play an important role in the boundary layer flow for controlling momentum and heat transfer for different fluid used as coating material for wire coating process. In view of the above motivation many researchers used the magnetic field as well as porous matrix for different Newtonian and non-Newtonian fluids [26–29]. Liu [30] investigated the second grade fluid in the presence of applied magnetic field. Viscoelastic fluid was used by Salem [31] in the presence of variable viscosity and thermal conductivity by utilizing shooting method over stretching sheet. Shah et al. [32] investigated the third grade in the absence of magnetic field for wire coating process. Bhukts et al. [33] investigated heat transfer effect on Oldroyd 8-constant fluid with wire coating analysis. Nayak et al. [19] studied wire coating analysis using MHD third grade fluid with temperature dependent viscosity. The same author [34] analyzed the unsteady free convective flow of a viscoelastic fluid with radiation and MHD effect through an inclined porous plate. Effect of chemical reaction and MHD on viscoelastic fluid flow over a stretching sheet embedded with porous medium was also investigated by Nayak [35]. Nayat et al. [36] analyzed the MHD flow of a viscoelastic fluid with effect of chemical reaction and porous medium.

Viscosity is one of the most important thermo-physical properties of a fluid. The variation of viscosity is no longer valid if the temperature variation during the flow is large. The variation of viscosity is significant in numerous applications such as in the process of hot rolling, wire drawing, glass fiber production, paper production, gluing of labels on hot bodies, drawing of plastic films, etc. Due to wide application of variation viscosity many researchers investigated the temperature dependent viscosity under various flow conditions [37].

In industries as well as technological process the thermal radiation, MHD and heat transfer problems have gained significant importance. Many production process occurs at high temperatures where knowledge of radiative heat transfer is very important for the design of the necessary equipment. Rhaman and Salahuddin [38] investigated the MHD flow of thermal radiation and heat transfer analysis with variable viscosity over an inclined plate.

In view of the above motivation, the objective of the present study is to analyze the wire coating process using coating material modeled as third grade fluids such as melt polymer and considering temperature dependent viscosity by utilizing Reynolds and Vogel’s models. In this context, the constitutive equations for velocity and temperature profiles are solved analytically by applying homotopy analysis method (HAM). The effect of emerging parameters are shown graphically and discussed in detail. The convergence of the method is also shown by calculating the h-curve which is now called convergence control parameter. The convergence, accuracy and efficiency of different optimization approaches are investigated. The calculations are carried out on a personal computer with 4GB RAM and 2.70GHz CPU. The code is developed using symbolic computing software MATHEMATICA [39]. The obtained results are also verified by utilizing a numerical technique so called ND-Solve method through graphically and numerically by calculating the absolute error. HAM is one of the most popular and frequently used method. Many researchers used this method to solve the nonlinear equations. Liao [40] applied this method for the solution of unsteady boundary layer flows caused by an impulsively stretching plate. The same author [41] investigated the boundary layer flows over a permeable stretching plate using HAM. Abbas bandy [42] gave the application of homotopy analysis method to nonlinear equations arising in heat transfer. This technique has also already been used for the solution of various problems [43–47]. Furthermore, for the sake of clarity the proposed method is also compared with Adomian Decomposition Method (ADM).

## Modeling of the problem

**Fig 2:**
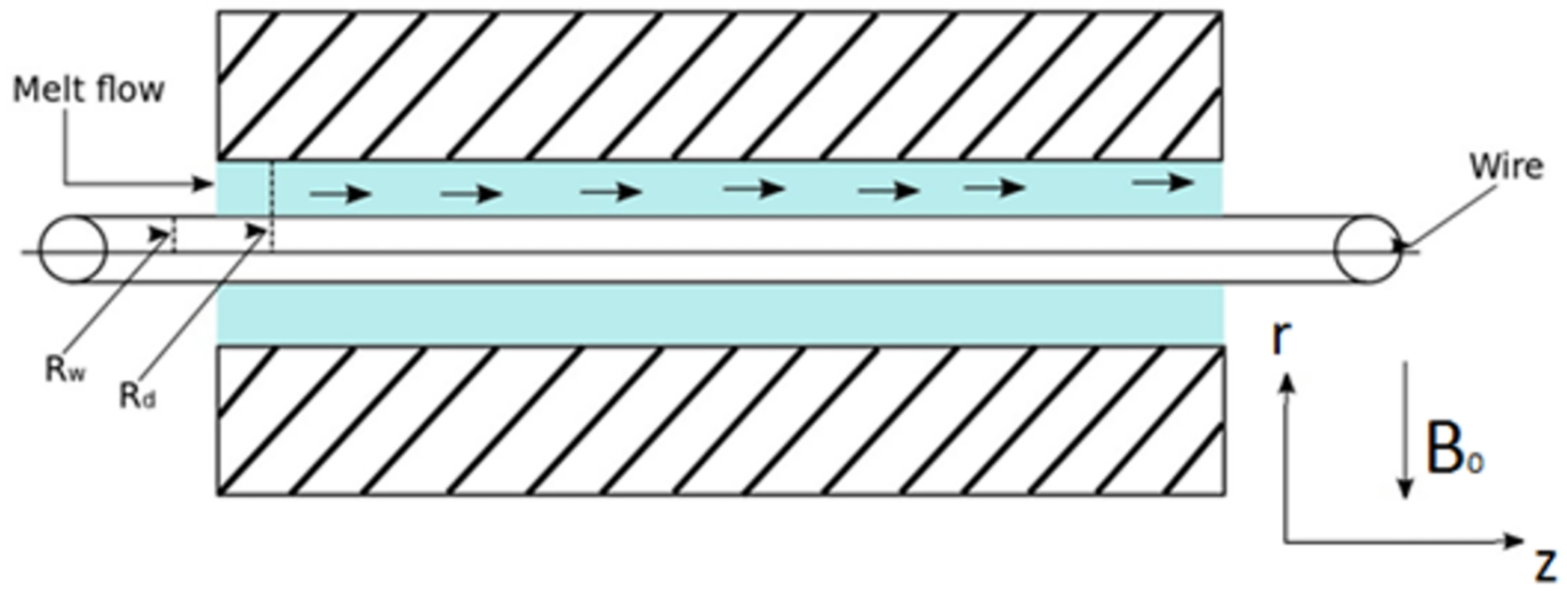
Wire coating process in a porous medium in a pressure type coating die.

The schematic diagram of the flow geometry is shown in Fig 2. The wire is dragged from the center of the die of finite length *L*, radius *R*_*d*_ and temperature *θ*_*d*_ which is filled with melt polymer satisfying the third grade fluid model like polyvinyl chloride (PVC) at a constant velocity *V*_*w*_ having temperature *θ*_*w*_ and radius *R*_*w*_ The fluid electrically conducting in the presence of applied magnetic field of strength *B*_0_ and acts upon a constant pressure gradient in the axial direction. The magnetic field is assumed to be normal to the flow. The induced magnetic field is negligible due to small Reynolds number. As a rresult the Lorentz force comes into play in the present set up which affects the coating process. Third grade fluid is used as melt polymer in the pressure type coating die while the die and wire are concentric. Cylindrical coordinate system is chosen in which r is taken normal to the flow and z-axis along the flow.

The flow is considered steady, laminar and axisymmetric.

Moreover, the structure of the die is important since it affects final product quality. Here in this analysis pressure type coating die is used. Let us avoid excessive shear stresses at the wire which may lead to elongation or frequent breakage of the wire during coating operation and also excessive wall shears tress which may result in un even and rough extruded coating. Nomenclature is given in Table 1.

**Table 1:** Nomenclature of the model.

*V*_*w*_	Velocity of the wire	*B*_0_	Strenght of uniform transverse magnetic field	$\vec{B}$	Magnetic field
*R*_*w*_	Radius of the wire	*c*_*p*_	Specific heat	β	Non-Newtonian parameter
*R*_*d*_	Radius of the die	*k*	Thermal conductivity	*M*	Magnetic field
*L*	Finite length of the die	ϕ	Dicisipation function	σ	Electric conductivity
θ	Temperature of the fluid	ψ	Stefan-Boltzmann coefficient	*R*	Radiation parameter
θ_*w*_	Wire temperature	γ	Mean absorption coefficient	χ	Reynolds model parameter
θ_*d*_	Flow temperature	η	Coefficient of viscosity	Ω	Vogel’s model parameter
ρ	Density of the fluid	$\vec{J}$	Current density		
*D*/*Dt*	Material derivative				
*H*	Radiative heat flux				

In view of the above frame of reference and assumptions the velocity, extras tress tensor and temperature fields are considered as [19].

$$\vec{w}=\left[0,0,u\left(r\right)\right],S=S\left(r\right),\theta =\theta (r).$$ (1)

Subject to the conditions

$$\begin{array}{l} u=V_{w},\theta =\theta_{w}\;\text{at}\;r=R_{w},\\ u=0,\theta =\theta_{d}\;\text{at}\;r=R_{d}. \end{array}$$ (2)

For third grade fluid the *S* is defined as

$$S=\eta A_{1}+\alpha_{1}A_{2}+\alpha_{2}A_{1}+\tau_{1}A_{2}+\tau_{2}\left(A_{1}A_{2}+A_{2}A_{1}\right)+\tau_{3}\left(trA_{2}\right)A_{1},$$ (3)

in which μ, is the coefficient of the viscosity of the fluid, *α*_1_, *α*_2_, *τ*_1_, *τ*_2_, *τ*_3_ are the material constant and *A*_1_, *A*_2_, *A*_3_ are line kinematic tensors.

$$A_{1}=L^{T}+L,A_{n}=A_{n-1}L^{T}+LA_{n-1}+\frac{DA_{n-1}}{Dt},n=2,3,$$ (4)

where *T* denotes transpose of a matrix.

The governing equations for an incompressible fluid are [5–12]

$$\nabla .\vec{w}=0,$$ (5)

$$\rho \frac{D\vec{w}}{Dt}=-\vec{\nabla }p+\vec{F}+\vec{J}\times \vec{B},$$ (6)

$$\rho c_{p}\frac{D\theta }{Dt}=k\nabla^{2}\theta +\phi -\frac{dH}{dr},$$ (7)

In above equations *ρ* is the density of the fluid, $\begin{array}{l} \frac{D}{Dt} \end{array}$ is the material derivative, *H* is the radiative heat flux, $\vec{J}$ is the current density, $\vec{B}$ is the total magnetic field, *C*_*p*_ is the specific heat, *k* is the thermal conductivity, *ϕ* is the dissipation function and $\vec{w}$ is the velocity vector.

A uniform magnetic field of strength is assumed to be applied in the positive radial direction normal to the wire i.e., along the retarding force per unit volume acting along z-axis is given by

$$\vec{J}\times \vec{B}=(0,0,-\sigma B_{0}^{2}u).$$ (8)

From Eqs (1)–(8) and above assumptions with the absence of pressure gradient we have

$$2\left(\tau_{2}+\tau_{3}\right)\frac{d}{dr}\left(r{\left(\frac{du}{dr}\right)}^{3}\right)+\frac{\eta }{r}\frac{d}{dr}\left(r\frac{du}{dr}\right)-\sigma B_{0}^{2}u=0,$$ (9)

The radiative heat flux is (se Brewster [48])

$$H=-\frac{4\psi \partial (\theta^{4})}{3ϒ\partial r},$$

Where *ψ* is the Stefan-Boltzmann constant *ϒ* is the mean absorption coefficient.

Expanding *θ*^4^ in a Taylor series about *θ*_*∞*_ and neglecting higher order terms [19]

$$\theta^{4}≅4\theta_{\infty }^{3}\theta -3\theta_{\infty }^{4},$$

The gradient of heat radiation is:

$$\frac{\partial H}{\partial r}=-\frac{16\theta_{\infty }^{3}}{3ϒ}\frac{\partial^{2}\theta }{\partial r^{2}}.$$ (10)

By incorporating the Eq (10), above equation becomes

$$k\left(\frac{d^{2}\theta }{dr^{2}}+\frac{1}{r}\frac{d\theta }{dr}\right)+\eta {\left(\frac{du}{dr}\right)}^{2}+2\left(\tau_{2}+\tau_{3}\right){\left(\frac{du}{dr}\right)}^{4}+\frac{16\theta_{\infty }^{3}}{3ϒ}\frac{\partial^{2}\theta }{\partial r^{2}}=0,$$ (11)

## Temperature dependent viscosity

### Reynold model

In case of Reymolds model the variable viscosity can be expressed as [19]

η=exp(−βΩθ)≈1−βΩθ, (12)

where Ω is viscosity parameter.

We introduce the following dimensionless parameters

$$\begin{array}{l} r^{*}=\frac{r}{R_{w}},u^{*}=\frac{u}{V},\beta =\tau_{2}+\tau_{3},\frac{R_{d}}{R_{w}}=\delta >1,\beta^{*}=\frac{\beta }{\eta \left(\frac{R_{w}^{2}}{V_{w}^{2}}\right)},M^{2}=\frac{\sigma B_{0}^{2}R_{w}}{\eta }\\ \;,\theta^{*}=\frac{\theta -\theta_{w}}{\theta_{d}-\theta_{w}},Br=\frac{\eta V_{w}^{2}}{k(\theta_{d}-\theta_{w})},\eta^{*}=\frac{\eta }{\eta_{0}}. \end{array}$$ (13)

In view of Eq (13), the above equations of momentum and energy become along with the corresponding boundary conditions

$$\left(1-\beta \Omega \theta \right)\left(r\frac{d^{2}u}{dr^{2}}+\frac{du}{dr}\right)+2\beta \left(3r\frac{d^{2}u}{dr^{2}}{\left(\frac{du}{dr}\right)}^{2}+{\left(\frac{du}{dr}\right)}^{3}\right)-\beta \Omega \frac{d\theta }{dr}\frac{du}{dr}-M^{2}ur=0,$$ (14)

u(1)=1,u(δ)=0, (15)

$$\left(1+R\right)\frac{d^{2}\theta }{dr^{2}}+\frac{1}{r}\frac{d\theta }{dr}+\left(1-\beta \Omega \theta \right)Br{\left(\frac{du}{dr}\right)}^{2}+2Br\beta {\left(\frac{du}{dr}\right)}^{4}=0,$$ (16)

θ(1)=0,θ(δ)=1. (17)

### Solution by homotopy analysis method (HAM)

Here we use the homotopy analysis method (HAM) to solve the nonlinear Eqs (14) and (16) corresponding to the boundary conditions given in Eqs (15) and (17) respectively.

The initial solution is obtained as:

$$u_{0}(r)=\frac{-r+\delta }{-1+\delta }\;\text{and}\;\theta_{0}(r)=\frac{r-1}{-1+\delta }.$$ (18)

The linear operator is selected as *L*_*f*_ and L_θ_:

$$L_{w}(u)=u\prime \prime \;\text{and}\;L_{\theta }(\theta )=\theta \prime \prime .$$ (19)

With the following properties:

$$L_{u}(c_{1}+c_{2}r)=0\;\text{and}\;{\text{L}}_{\theta }(c_{3}+c_{4}r)=0,$$ (20)

where *c*_*i*_(*i* = 1−4) are constants.

The zeroth order problem:

$$(1-p)L_{u}\left[u(r;p)-u_{0}(r)\right]=p\hbar_{u}N_{u}\left[u(r;p)\right],$$ (21)

$$(1-p)L_{\theta }\left[\theta (r;p)-\theta_{0}(r\right]=p\hbar_{\theta }N_{\theta }\left[u(r;p),\theta (r;p)\right].$$ (22)

u(1;p)=1,θ(1;p)=0, (23)

u(δ;p)=0,θ(δ;p)=1, (24)

In above equations $\hbar_{u}$ and $\hbar_{\infty }$ convergence control parameters, *p* ∊ [0,1] is an embedding parameter while the non-linear operator is chosen as:

$$\begin{array}{l} N_{u}\left[u(r;p)\right]=\left(1-\beta \Omega \theta \right)\left(r\frac{\partial^{2}u(r;p)}{\partial r^{2}}+\frac{\partial u(r;p)}{\partial r}\right)+2\beta \left(3r\frac{\partial^{2}u(r;p)}{\partial r^{2}}{\left(\frac{\partial u(r;p)}{\partial r}\right)}^{2}+{\left(\frac{\partial u(r;p)}{\partial r}\right)}^{3}\right)\\ -\beta \Omega \frac{\partial \theta (r;p)}{\partial r}\frac{\partial u(r;p)}{\partial r}-M^{2}ur=0, \end{array}$$ (25)

$$\begin{array}{l} N_{\theta }\left[u(r;p),\theta (r;p)\right]=\left(1+R\right)\left(\frac{\partial^{2}\theta (r;p)}{\partial r^{2}}+\frac{1}{r}\frac{\partial \theta (r;p)}{\partial r}\right)+\left(1-\beta \Omega \theta \right)Br{\left(\frac{\partial u(r;p)}{\partial r}\right)}^{2}+\\ 2Br\beta {\left(\frac{\partial u(r;p)}{\partial r}\right)}^{4}. \end{array}$$ (26)

For *p* = 0 and *p* = 1, the zeroth order deformation problem yields respectively:

$$u(r;0)=u_{0}(r)\;\text{and}\;u(r;1)=u(r),$$ (27)

$$\theta (r;0)=\theta_{0}(r)\;\text{and}\;\theta (r;1)=\theta (r).$$ (28)

As *p* vvaries fromm0 to 1, *u* (*r*, *p*) varies from *u*_0_ (*r*) too *u* (*r*) and *θ*_0_ (*r*) to *θ* (*r*)

Expanding *u*(*r*;*p*) and *T*(*r*;*p*) in Taylor’s series of *p*. Due to taylor’s series and Eq (28), we have

$$u(r;p)=u_{0}(r)+\sum_{m=1}^{\infty }u_{m}(r)p^{m},$$ (29)

$$\theta (r;p)=\theta_{0}(r)+\sum_{m=1}^{\infty }\theta_{m}(r)p^{m}.$$ (30)

Where

$$u_{m}(r)={\frac{1}{m!}\frac{\partial u(r;p)}{\partial r}|}_{p=0}\;\text{and}\;\theta_{m}(r)={\frac{1}{m!}\frac{\partial \theta (r;p)}{\partial r}|}_{p=0}.$$

The m^th^ order deformation problems

$$L_{u}\left[u_{m}(r)-\chi_{m}u_{m-1}(r)\right]=\hbar_{u}R_{m}^{u}(r),$$ (31)

$$L_{\theta }\left[\theta_{m}(r)-\chi_{m}\theta_{m-1}(r)\right]=\hbar_{\theta }R_{m}^{\theta }(r).$$ (32)

$$u_{m}(1)=u_{m}(\delta )=0,$$ (33)

$$\theta_{m}(1)=\theta_{m}(\delta )=0.$$ (34)

In which

$$R_{m}^{u}(r)=\left(1-\beta \Omega \theta \right)\left(r\frac{d^{2}u_{m-1}}{dr^{2}}+\frac{du_{m-1}}{dr}\right)+6\beta r\overset{m-1}{\underset{k=0}{\sum }}\overset{k}{\underset{i=0}{\sum }}\frac{du_{m-1-k}}{dr}\frac{du_{k-i}}{dr}\left[\frac{d^{2}u_{m-1}}{dr^{2}}\right]+2\beta \overset{m-1}{\underset{k=0}{\sum }}\overset{k}{\underset{i=0}{\sum }}\overset{i}{\underset{j=0}{\sum }}\frac{du_{m-1-k}}{dr}\frac{du_{k-i}}{dr}\frac{du_{i-j}}{dr}-\beta \Omega \frac{d\theta_{m-1}}{dr}\frac{du_{m-1}}{dr}-rM^{2}u_{m-1},$$ (35)

$$R_{m}^{\theta }(r)=\left(1+R\right)\left(r\frac{d^{2}\theta_{m-1}}{dr^{2}}+\frac{d\theta_{m-1}}{dr}\right)+r\left(1-\beta \Omega \theta \right)Br\overset{m-1}{\underset{k=0}{\sum }}\overset{k}{\underset{i=0}{\sum }}\frac{du_{m-1-k}}{dr}\frac{du_{k-i}}{dr}+2r\beta Br\overset{m-1}{\underset{k=0}{\sum }}\overset{k}{\underset{i=0}{\sum }}\overset{i}{\underset{j=0}{\sum }}\overset{j}{\underset{l=0}{\sum }}\frac{du_{m-1-k}}{dr}\frac{du_{k-i}}{dr}\frac{du_{i-j}}{dr}\frac{du_{j-l}}{dr}.$$ (36)

where $\chi_{m}=\left\{ \begin{array}{l} 0,\;\text{if}\;\text{p}\leq \text{1}\\ 1,\;\text{if}\;\text{p}>\text{1} \end{array}\right.$

The linear non-homogeneous problems (31) and (32) corresponding to the boundary conditions (33) and (34) can be easily solved by using MATHEMATICA in order m = 1,2,3,…

### Vogel’s model

In this case, the temperature dependent viscosity is taken as [19]

$$\eta =\eta_{0}\text{exp}(\frac{H}{F+\theta }-\theta_{w}),$$ (37)

After using the expansion we have

$$\eta =\Omega (1-\frac{H}{F^{2}}\theta ),$$ (38)

where $\Omega =\eta_{0}\text{exp}(\frac{H}{F}-\theta_{w})$ and H, F are viscosity parameters associated with Vogel’s model.

So the non-dimensional momentum and energy equations with boundary conditions omitting steriks are

$$\Omega \left(1-\frac{H}{F^{2}}\theta \right)\left(r\frac{d^{2}u}{dr^{2}}+\frac{du}{dr}\right)+2\beta \left(3r\frac{d^{2}u}{dr^{2}}{\left(\frac{du}{dr}\right)}^{2}+{\left(\frac{du}{dr}\right)}^{3}\right)-\left(\frac{\Omega Η}{F^{2}}\right)\frac{d\theta }{dr}\frac{du}{dr}-M^{2}ur=0,$$ (39)

u(1)=1,u(δ)=0, (40)

$$\left(1+R\right)\frac{d^{2}\theta }{dr^{2}}+\frac{1}{r}\frac{d\theta }{dr}+\Omega \left(1-\frac{H}{F^{2}}\theta \right)Br{\left(\frac{du}{dr}\right)}^{2}+2Br\beta {\left(\frac{du}{dr}\right)}^{4}=0,$$ (41)

θ(1)=0,θ(δ)=1. (42)

### Solution by homotopy analysis method (HAM)

Eqs (39) and (41) with boundary conditions (40) and (42) are solved analytically by applying the homotopy analysis method as mentioned in Reynolds model case.

### Convergence of the HAM solution

**Fig 3:**
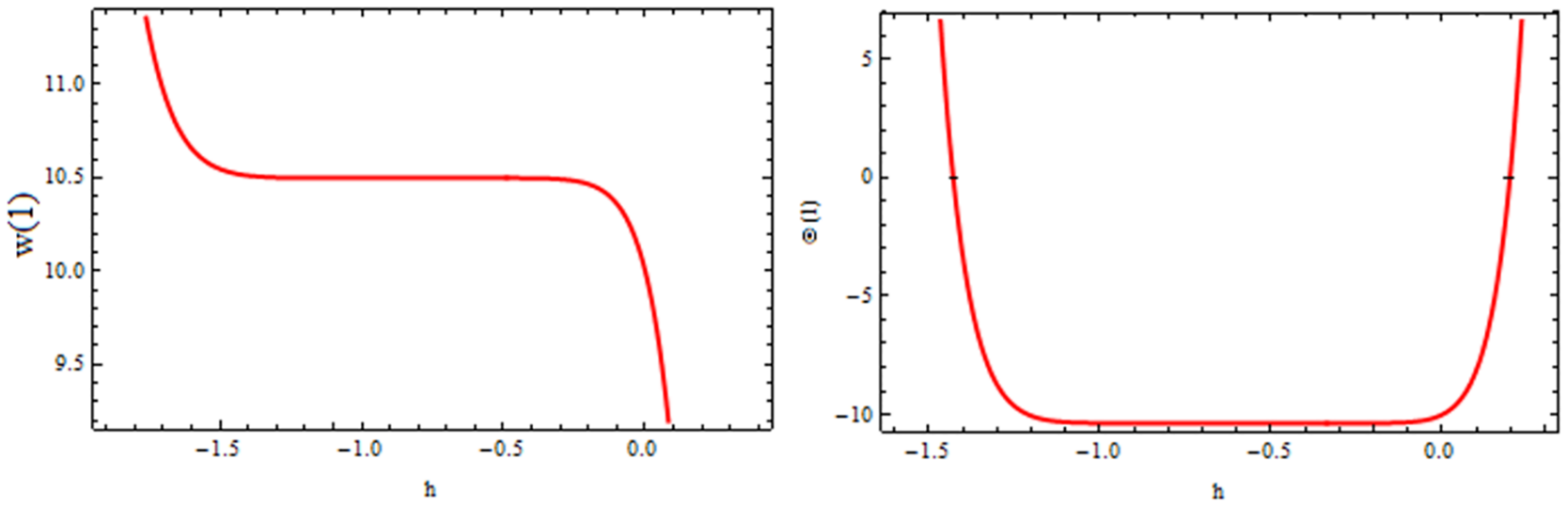
The ℏ- curve of velocity and temperature profiles for 20^th^-order approximation.

**Fig 4:**
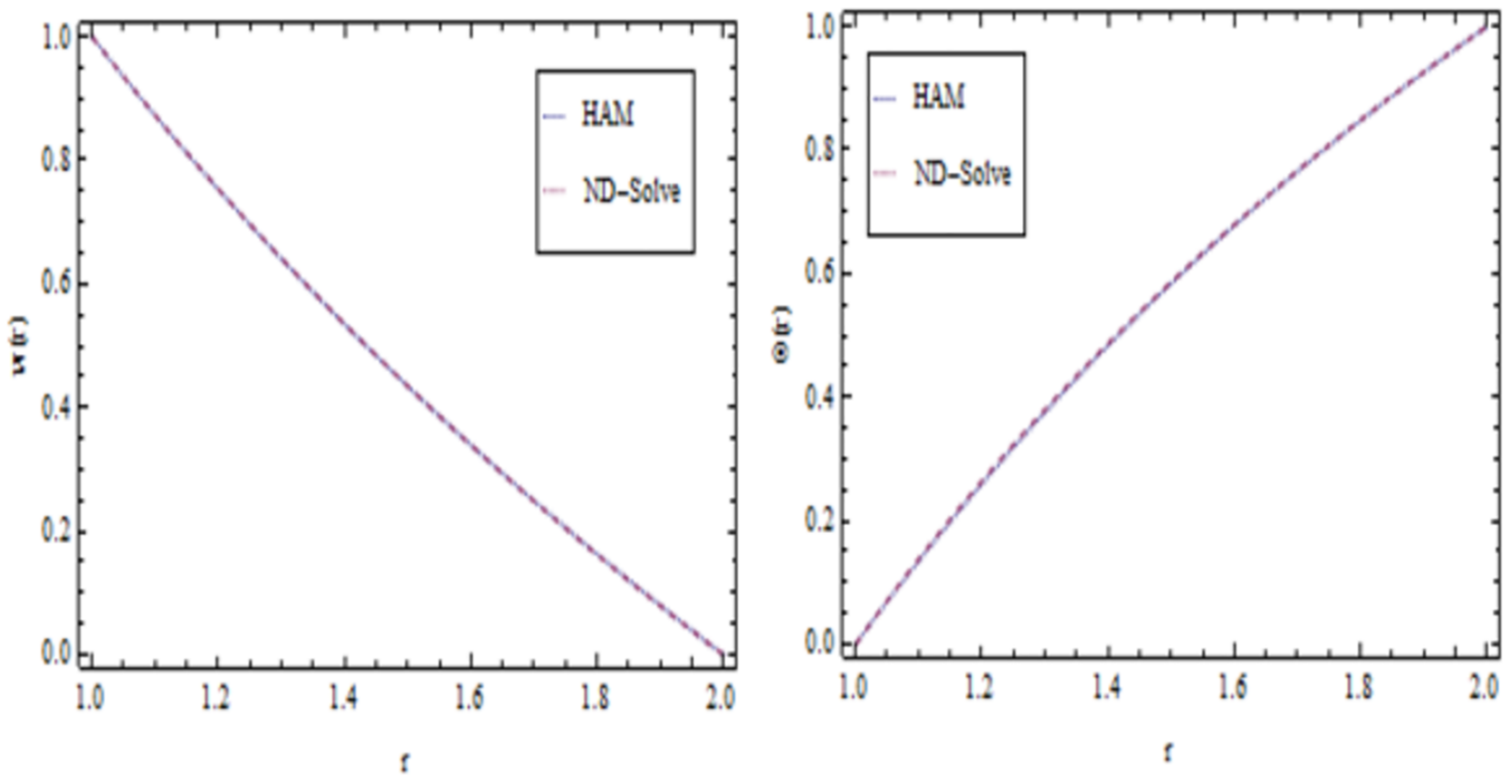
Comparison of HAM and ND-Sole methods.

It should be noted that the series solution (39) and (41) contain the auxiliary parameters $\hbar_{w}$ and $\hbar_{\theta }$ that provide a simple way to adjust and control the convergence of the series solution. Through ℏ-curves, it is directly possible to choose an appropriate range for $\hbar_{w}$ and $\hbar_{\theta }$ which ensures the convergence of series solutions. The calculations are carried out on a personal computer with 4GB RAM and 2.70 GHz CPU. The code is developed using symbolic computing software MATHEMATICA [39]. To see the range of admissible values of these parameters, the curves of $\hbar_{w}$ and $\hbar_{\theta }$ are plotted in Figs 3 and 4 given by 20^th^-order approximation which take approximately less than a minute in execution. The suitable range for $\hbar_{w}$ and $\hbar_{\theta }$ are $-1.1\leq \hbar_{w}\leq -0.9$ and $-1.4\leq \hbar_{\theta }\leq 0$ respectively. The convergence of the method is also clear from Tables 2 and 3 by calculating the pade-approximation.

**Table 2:** The homotopy-pade approximation of *w*(1) and *θ*(1) for Reynlods model when *χ* = 6, *R* = 2, *Br* = 10, *M* = 0.1, *β* = 0.2.

r	*w*(1)	*θ*(1)
[2/2]	-0.451024	-0.654021
[3/3]	-0.452521	-0.653145
[4/4]	-0.452365	-0.653541
[5/5]	-0.452630	-0.653640
[6/6]	-0.452315	-0.654612
[7/7]	-0.452001	-0.654441

**Table 3:** The homotopy-pade approximation of *w*(1) and *θ*(1) for Vogel’s model when *Ω* = 6, *R* = 2, *Br* = 13, *M* = 0.1, *β* = 0.2.

r	*w*(1)	*θ*(1)
[2/2]	-0.7624392	-0.663219
[3/3]	-0.7621111	-0.6639111
[4/4]	-0.7636667	-0.6646667
[5/5]	-0.7633333	-0.6645953
[6/6]	-0.7621012	-0.6634444
[7/7]	-0.7621142	-0.6633568

## Validation of the results

The convergence of the method is also necessary to check the reliability of the methodology. The convergence of the obtained series is shown in Fig 3. The current results are also compared with a numerical method so called ND-Solve method, and an outstanding correspondence is seen to exist between the two sets of data as revealed in Fig 4. Furthermore, the comparison of the present method with Adomian Decomposition Method (ADM) and ND-Solve is also given in Table 4.

**Table 4:** Numerical comparison for velocity profile given in Eq (39) using HAM, ND-Sole methods and ADM *Ω* = 5, *R* = 2, *Br* = 10, *M* = 0.1, *β* = 0.2.

r	HAM	ND-solve	ADM
1.0	1	1	1
1.2	0.66070791111	0.66070791111	0.66070791123
1.4	0.41585866667	0.41585866667	0.41585866665
1.6	0.23559573333	0.23559573333	0.23559573322
1.8	0.10125404444	0.10125404444	0.10125404443
2.0	0	1.17763568×10^−14^	1.015426227×10^−13^

## Results and discussion

Wire coating process is analyzed in a pressure type coating die using viscoelastic third grade fluid with variable viscosity. For this purpose Reynolds model and Vogel’s model are used. An approximate analytical solution has been obtained by applying Homotopy Analysis Method (HAM). For the sake of clarity the results are also verified by Adomian Decomposition Method (ADM) and ND-Solve method graphically and numerically. The effects of emerging parameters involved in the solution especially the non-Newtonian parameter β Reynolds model viscosity parameter χ Vogel’s model viscosity parameter Ω thermal radiation parameter *R* and Brinkman number *Br* are discussed in detail.

The flow and heat transfer phenomena occurring inside the wire coating dies determine the quality of the coated wire produced.

### Reynolds model

**Fig 5:**
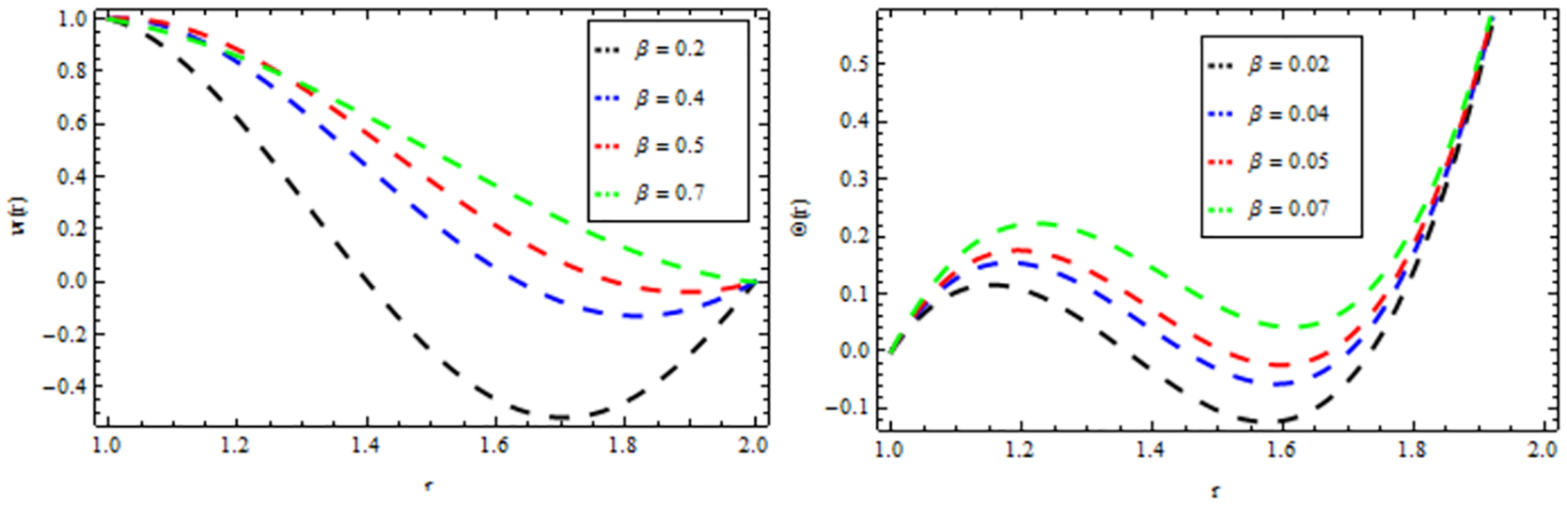
Velocity and temperature distribution showing the effect of viscoelastic parameter *β* when *χ* = 6, *R* = 2, *Br* = 10, *M* = 0.1.

**Fig 6:**
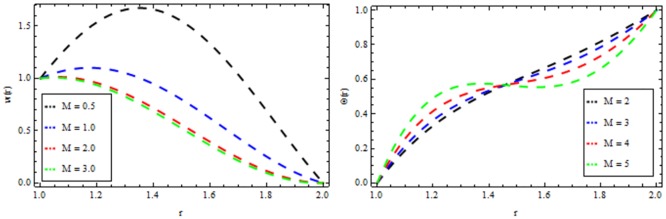
Velocity and temperature distribution showing the effect of magnetic parameter *M* when *χ* = 6, *R* = 2, *Br* = 10, *β* = 0.2.

**Fig 7:**
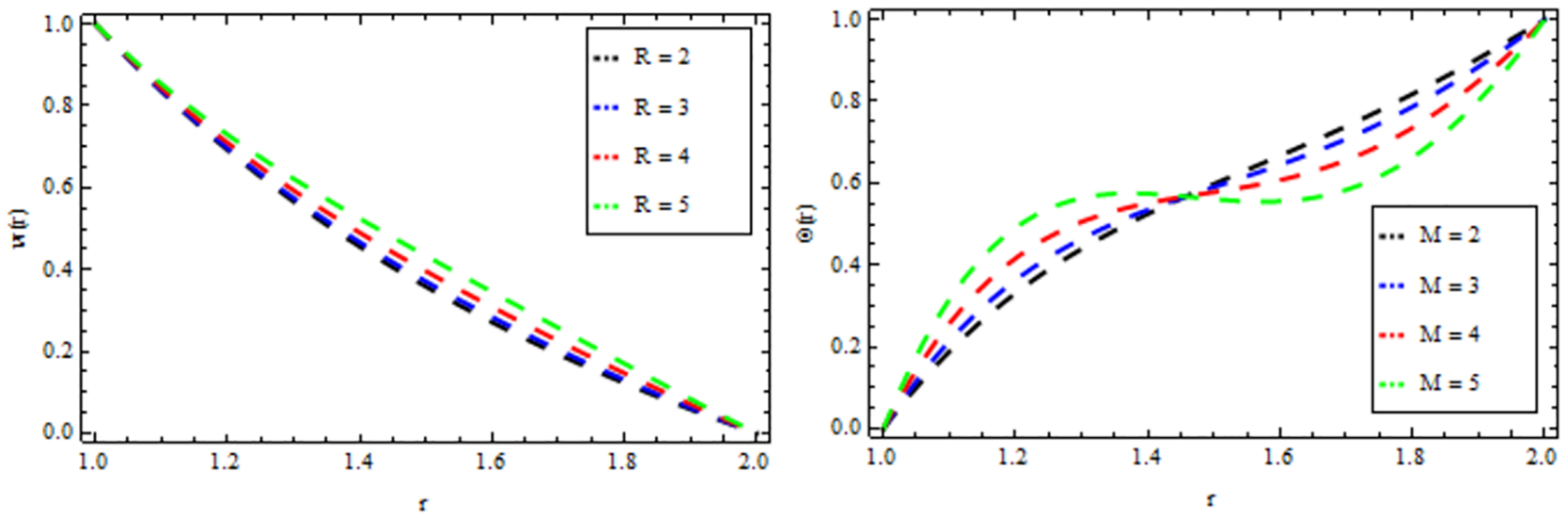
Velocity and temperature distribution showing the effect of thermal radiation parameter *R* when *χ* = 6, *M* = 0.1, *Br* = 10, *β* = 0.2.

**Fig 8:**
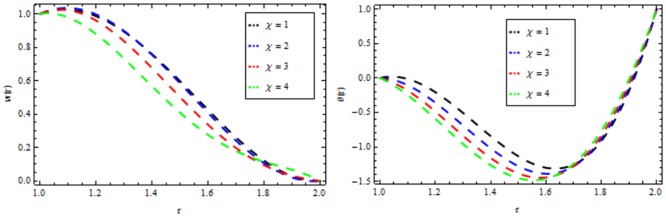
Velocity and temperature distribution showing the effect of Reynolds viscosity *χ* when *R* = 2, *M* = 0.1, *Br* = 10, *β* = 0.2.

**Fig 9:**
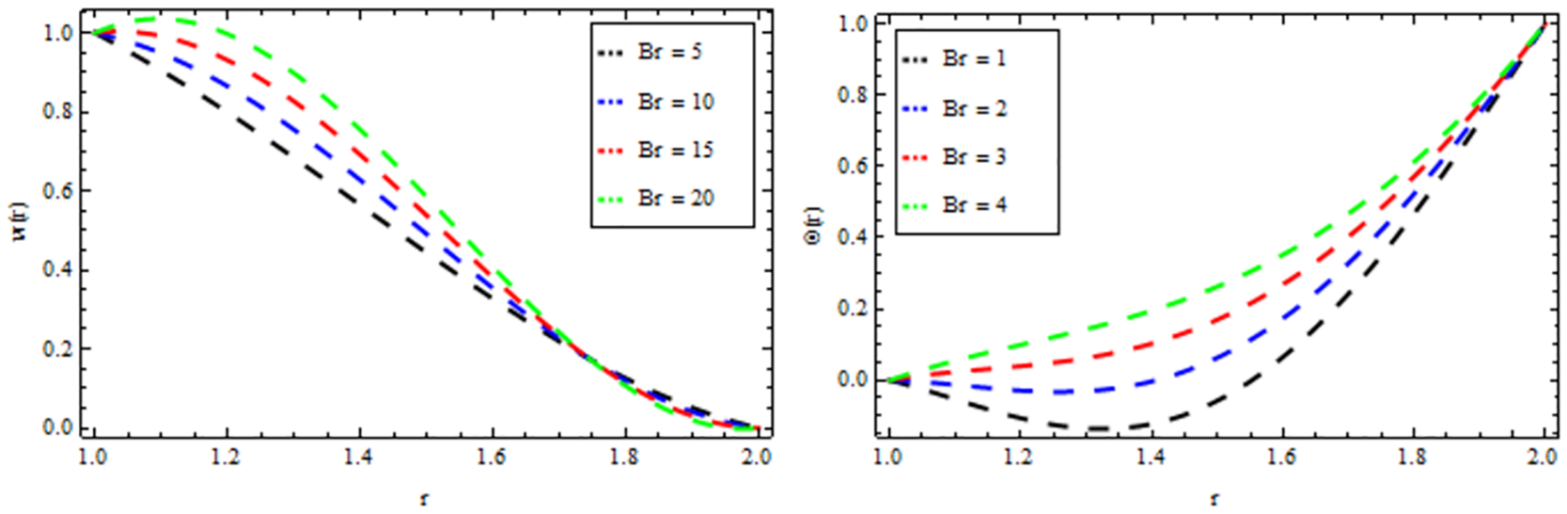
Velocity and temperature distribution showing the effect of Brinkman number *Br* when *χ* = 6, *M* = 0.1, *Br* = 10, *β* = 0.2.

The effect of non-Newtonian parameter β of third grade fluid on the velocity and temperature profiles is shown in Fig 5 while taking the other parameters fixed. From this figure it is observed that the velocity and temperature profiles increase inside the coating die as the non-Newtonian parameter increases. The effect of magnetic parameter *M* on the velocity and temperature profiles is depicted in Fig 6. It is observed that the velocity profile decreases as the magnetic parameter increases and highly temperature is observed within the layer 1≤*r*≤1.5 and thereafter, the temperature falls. The effect of thermal radiation parameter *R* on the velocity and temperature profile is shown in Fig 7. It is observed that the thermal radiation accelerates the velocity and temperature of the polymer. It is interesting to note that for high values of thermal radiation parameter the coating process is more significant near the surface of the wire which is also acceptable for manufacturing. Fig 8 presents the effect of viscosity parameter χ on the velocity and temperature profiles. It has been found that the velocity decreases while temperature increases. The variation of velocity and temperature profiles for different of Brinkman number *Br* is shown in Fig 9. It is observed that velocity and temperature profile increases with increasing *Br* From these simulations, it is observed that in the wire coating process, the relative measure of viscous heating with heat conducted and the Brinkman number, enhances the velocity and temperature which in turn accelerates the coating process.

### Vogel’s model

**Fig 10:**
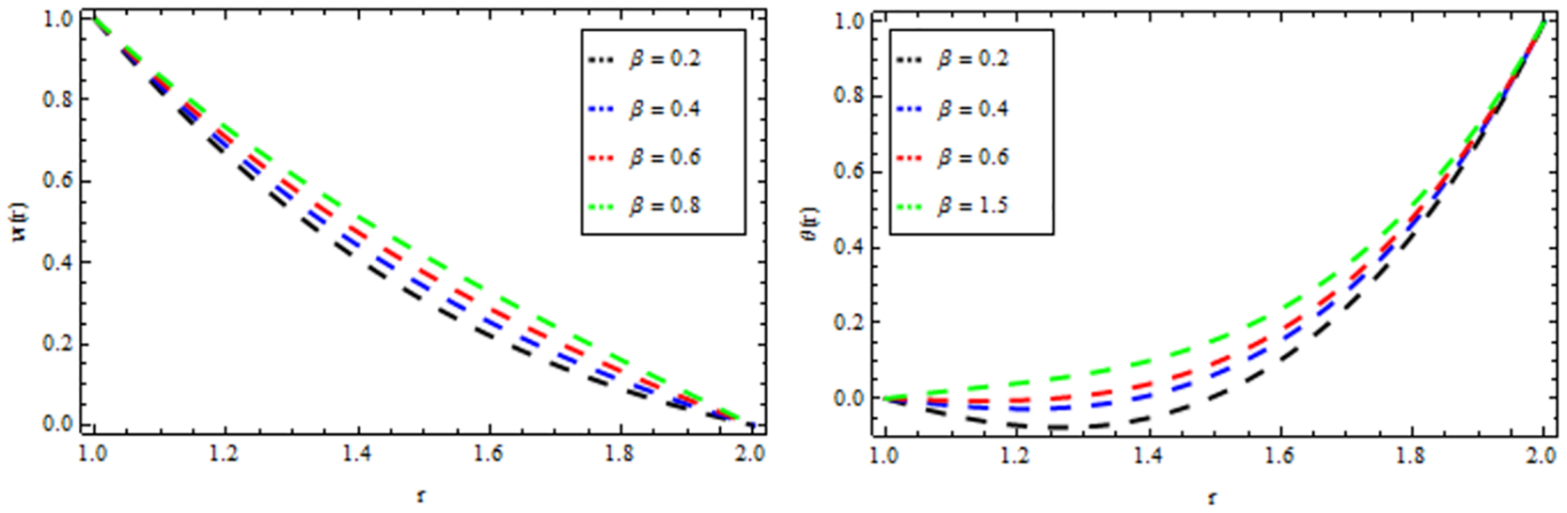
Velocity and temperature distribution showing the effect of viscoelastic parameter *β* when *Ω* = 6, *R* = 2, *Br* = 13, *M* = 0.1.

**Fig 11:**
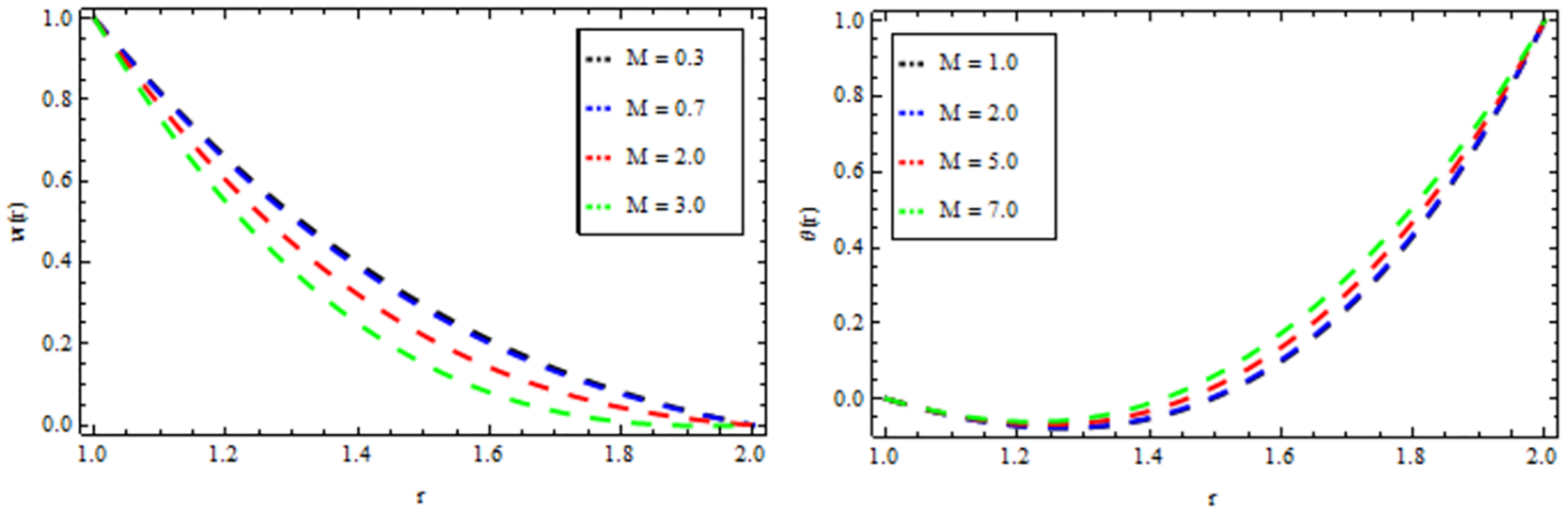
Velocity and temperature distribution showing the effect of magnetic parameter *M* when *Ω* = 6, *R* = 2, *Br* = 13, *β* = 0.2.

**Fig 12:**
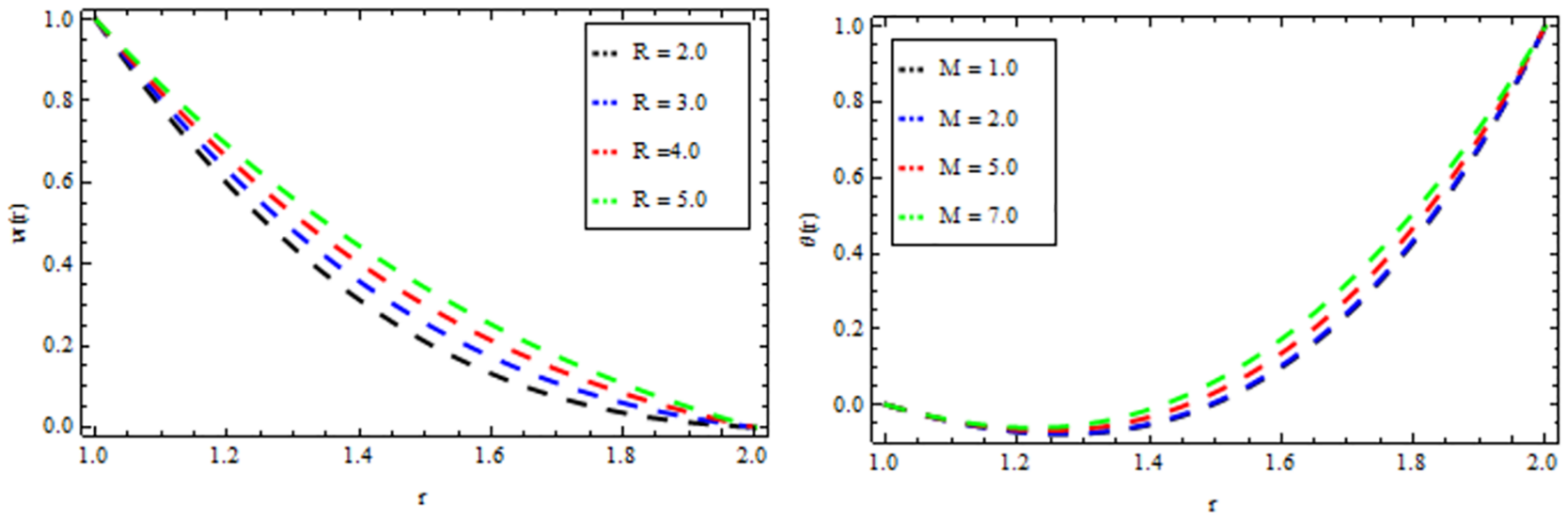
Velocity and temperature distribution showing the effect of thermal radiation parameter *R* when *Ω* = 6, *M* = 0.1, *Br* = 13, *β* = 0.2.

**Fig 13:**
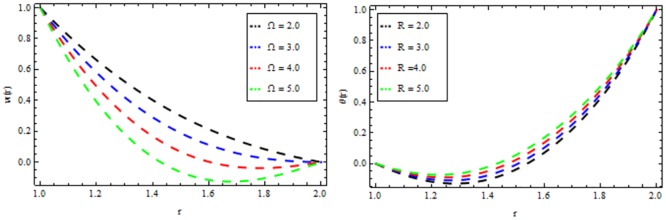
Velocity and temperature distribution showing the effect of Reynolds viscosity Ω when *R* = 2, *M* = 0.1, *Br* = 13, *β* = 0.2.

**Fig 14:**
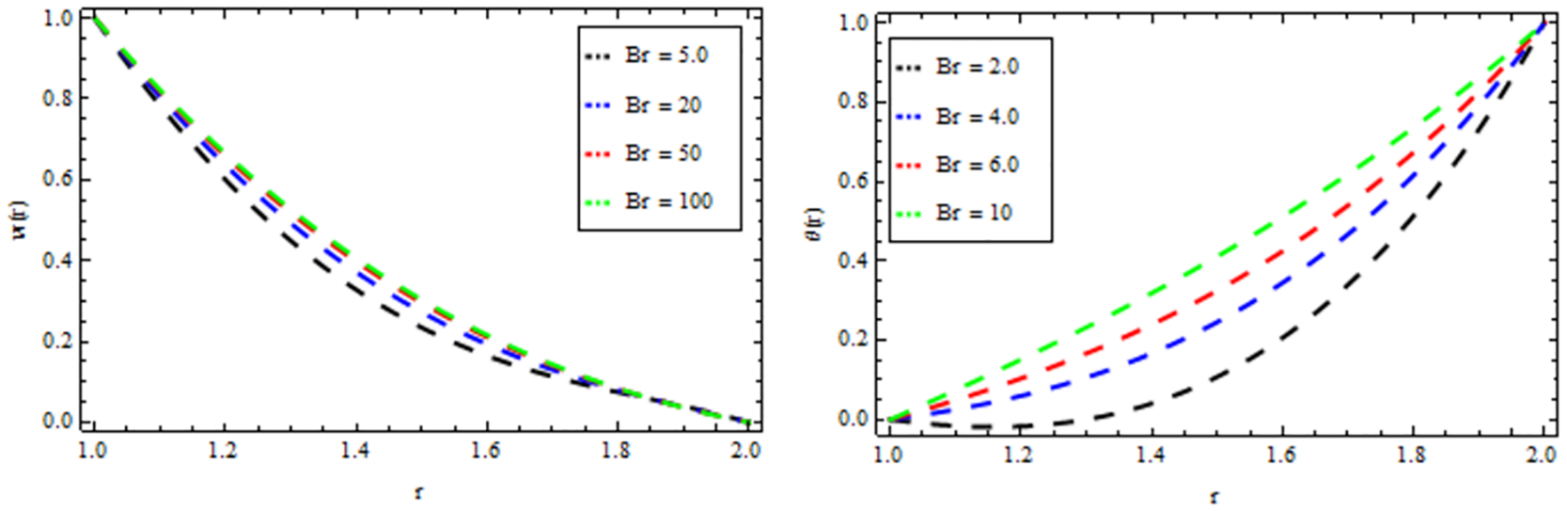
Velocity and temperature distribution showing the effect of Brinkman number *Br* when *Ω* = 6, *M* = 0.1, *Br* = 0.2, *R* = 2.

Figs 10–14 present the velocity and temperature profiles for several of non-Newtonian parameter property along with variable viscosity.

In Fig 10, we noticed that the velocity and temperature profile increases with an increase in non-Newtonian parameter β. Fig 11 depicts the variation of velocity and temperature profiles for different values of magnetic parameter *M*. It is clear that the velocity of the fluid decrease while the temperature profiles increases. Thermal radiation parameter accelerates the coating faster since it accelerates the velocity and temperature inside the melt polymer as shown in Fig 12. The effect of Vogel’s viscosity parameter Ω on the velocity and temperature profile is shown in Fig 13. It is found that the velocity and temperature profiles increase with the increasing value of Vogel’s viscosity parameter Ω Further, it is observed from Fig 14 that the velocity and temperature distribution increases with an increase in Brinkman number *Br*.

## Conclusion

In this study the radiative melt polymer satisfying the third grade fluid model is used for wire coating analysis. The effect of pertinent parameters involved in the solution is presented through graphically and numerically. Reynolds and Vogel’s models are used for the variable viscosity.

### Reynolds model

It is investigated that the non-Newtonian parameter of the viscoelastic third grade accelerates the velocity and temperature of the melt polymer. The magnetic parameter leads to increase the temperature in the entire flow domain. The presence of force due to application of moderately large magnetic field may contribute to nonlinearity of velocity variation. An increase in the thermal radiation parameter and Brinkman number accelerate the velocity and temperature of the melt polymer so as to make the process faster.

### Vogel’s model

In the presence of applied magnetic field the velocity of the fluid decreases. Thermal radiation parameter accelerates the coating faster since it accelerates the velocity and temperature inside the melt polymer. It is also investigated the viscous heating and viscoelastic property of the melt polymer accelerate the fluid temperature near the surface of the wire and it is counter productive near the inner surface of the die. The viscoelastic flows are full of instabilities such as in the flows of extrusion dying as claimed by Nhan-Phan-Thien which is compatible with the present study.
